# Individual and neighbourhood socioeconomic status increase risk of avoidable hospitalizations among Canadian adults: A retrospective cohort study of linked population health data

**DOI:** 10.23889/ijpds.v5i1.1351

**Published:** 2020-09-01

**Authors:** LE Wallar, LC Rosella

**Affiliations:** 1 Dalla Lana School of Public Health, 155 College St Room 500, University of Toronto, Toronto, ON M5T 3M7

## Abstract

**Introduction:**

Avoidable hospitalizations refer to acute care use for conditions that should normally be managed in primary care settings. Lower socioeconomic status that is often measured using area-based indicators (e.g. median household income) has been shown to increase risk of avoidable hospitalizations. However, both area- and individual-level socioeconomic status can contribute to hospitalization risk, but previous data limitations have prevented separate analyses. Further, the joint effect of individual and neighbourhood socioeconomic status has not been established in the Canadian population. To address this, this study links individual-level household income and neighbourhood-level material deprivation data within a population-based Canadian cohort.

**Objectives:**

To determine the individual and joint effect of individual-level household income and neighbourhood-level material deprivation on risk of hospitalization for a set of chronic ambulatory care sensitive conditions using linked health survey, hospital discharge, and census-derived data.

**Methods:**

A pooled cohort was created by linking sociodemographic and health information from eight cycles of the Canadian Community Health Survey (2000/2001 - 2010) to hospital discharge records and Canadian Marginalization Indices (2001, 2006) (N = 354,595). The primary outcome variable was risk of index hospitalization with a primary diagnosis of angina, asthma, congestive heart failure, chronic obstructive pulmonary disease, diabetes, epilepsy, or hypertension. The primary exposure variable was joint individual-level national household income quintile and neighbourhood-level material deprivation quintile. Relative risk (RR) was estimated by constructing modified Poisson regression models with robust error variance.

**Results:**

In fully adjusted models with income and deprivation considered separately, individuals in the lowest household income quintile and highest material deprivation quintile were at increased risk of hospitalization (Income RR: 1.82 (95% CI 1.56-2.13) Deprivation RR: 1.67 (1.44-1.95)). When income and deprivation were jointly considered, those with low individual income living in high deprivation neighbourhoods were at greatest risk of hospitalization (RR 1.83 (95% CI 1.63 - 2.05)).

**Conclusion:**

Both individual income and neighbourhood deprivation separately and jointly increase risk of avoidable hospitalizations. Additional research is needed to understand their mechanisms of action. However, both levels should be considered when designing effective policies and interventions to reduce avoidable hospitalizations.

## Introduction

Avoidable hospitalizations broadly refer to hospitalizations for a set of conditions for which timely and accessible primary care interventions exist [1]. In the Canadian context, avoidable hospitalizations specifically refer to acute care hospitalizations of individuals aged 0-74 years for angina, asthma, congestive heart failure (CHF), chronic obstructive pulmonary disease (COPD), epilepsy, and hypertension where the patient is discharged alive [2]. Other international definitions may include acute (e.g. cellulitis, dehydration) and vaccine-preventable conditions (e.g. influenza, measles) as well as age restrictions [3].

Avoidable hospitalizations are an important indicator of health system performance that are used by multiple international health systems [2, 4, 5]. Monitoring increases or disparities in avoidable hospitalization rates can signal the need for better primary care delivery and management. Although Canada has a universal health care system where medically necessary services are free at the point of care, demographic, socioeconomic, and geographic disparities in avoidable hospitalizations persist [6]. Avoidable hospitalizations also represent health resource inefficiencies [11, 6, 7]. In Canada, cost estimates for avoidable hospitalizations are $416 million per year (2006 estimate) [6]. Identification and action on modifiable risk factors for avoidable hospitalizations can improve population health and health system performance as well as reduce unnecessary costs.

There are a number of risk factors for avoidable hospitalizations [8-12]. In particular, low socioeconomic status (SES) has been shown to be associated with higher risk of avoidable hospitalizations across different SES measures (e.g. income [13, 14], education [15, 16], deprivation [17, 18]). In the Canadian context, SES disparities among both adult and pediatric populations have been described [66, 19]. While this association is well established, a continued focus on SES as a risk factor is important to address other unknowns including mechanisms of action, effective interventions and their entry points, and impact of individual and neighbourhood SES on risk of avoidable hospitalization. From a policy perspective, variation in avoidable hospitalizations by SES, particularly in a universal health care system, suggest important changes are needed to ensure health needs are equitably met.

However, many prior studies of the effect of SES on avoidable hospitalizations have used area-based SES information (e.g. median area household income, area deprivation) that lacks the granularity of individual level information. Other studies have not used appropriate regression for rate outcomes, have limited adjustment for potentially important confounding variables such as health behaviours, sociodemographics, and geography, or have presented model results that are not optimized to interpret SES effects as they adjust for other non-confounding variables. Further, heterogeneous definitions of SES exposures and avoidable hospitalization outcomes limit generalizability of findings. Many of these limitations stem from the fact that SES and other important confounding variables are not routinely collected as part of medical records or hospital billing data.

To address this, this study uses a large, national longitudinal database of linked health survey and hospital discharge records to determine the effect of both individual-level and neighbourhood-level SES on prospective risk of avoidable hospitalizations. The objective of this study was to determine the individual and joint effect of individual-level household income and neighbourhood-level material deprivation on risk of hospitalization for a chronic ambulatory care sensitive condition (ACSC), while controlling for potential confounding effects of other individual-level variables. This study contributes to the existing literature on the effects of SES on risk of avoidable hospitalizations by 1) examining the effects of both individual and neighbourhood SES separately and jointly on risk of avoidable hospitalizations, 2) directly estimating risk using time-to-event data, 3) controlling for a number of demographic and behavioural variables at the individual level that are not commonly available in hospitalization datasets, and 4) using the largest Canadian population-based cohort to examine these effects to date.

## Methods

A retrospective cohort study of eligible Canadian Community Health Survey (CCHS) respondents was conducted at the national level from 2000-2013. Exposure status was ascertained at the time of interview, and respondents were followed until an index outcome event, in-hospital death, or end of study (March 31, 2013). 

### Data sources

The CCHS is a national cross-sectional survey of population health status and its demographic, social, behavioural, and clinical determinants, and is administered by Statistics Canada [20]. The target population is Canadian youth and adults, 12 years of age and older, excluding those living on Aboriginal reserves and settlements, those living in select remote regions of Quebec and Nunavut, full-time military members, and institutionalized individuals. These exclusions constitute <3% of the target population [20]. Eight CCHS general population health survey cycles corresponding to survey years 2000-2001, 2003, 2005, 2007, 2008, 2009, 2010, and 2011 were used. For survey cycles 2005-2010, data files with imputed household income information were made available by Statistics Canada and used in this study [21].

The Discharge Abstract Database (DAD) is a national database of demographic, administrative, and clinical data relating to hospital inpatient discharges and day surgery interventions that is maintained by the Canadian Institute of Health Information [22]. All provinces and territories, excluding Quebec, submit information to the DAD, representing ~75% of all hospital separations in Canada. Data was available for fiscal year (FY) 1999/2000 - 2012/2013.

The Canadian Marginalization Index (CanMarg) is a census-based measure of socioeconomic status at the dissemination area (DA)-level [23]. DAs are the smallest standard geographic area for which census information is made publically available, representing 400-700 individuals [24]. In this study, the 2001 and 2006 CanMarg indices were used (Available at http://www.ontariohealthprofiles.ca/onmargON.php).

### Cohort creation

#### Linking CCHS and CanMarg information

Six-digit CCHS respondent postal codes were linked with DA identifiers using three versions of Statistics Canada Postal Code Conversion Files Plus (PCCF+) [25]. CCHS cycles 2000/2001 and 2003 were linked to PCCF+ Version 4J (2001 population weight), cycles 2005-2010 were linked to PCCF+ Version 5K (2006 population weight), and cycle 2011 was linked to PCCF+ Version 6D (2011 population weight). DA identifiers were then used to link CCHS cycles 2000/2001 and 2003 to CanMarg 2001 and CCHS cycles 2005-2011 to CanMarg 2006 information. 

#### Linking CCHS and DAD information

The eight CCHS survey cycles were pooled and deterministically linked to hospital separation records in the DAD for fiscal years 1999/2000 – 2012/2013 using unique household-person identifiers, retaining all CCHS respondents regardless of hospitalization status. Linkage was done in two steps. First, CCHS survey data was linked to intermediary merge keys using unique household-person identifiers creating respondent-key observations. These merge keys contained hospital transaction identification numbers needed for linkage to DAD information. Second, respondent-keys were linked to hospitalization records using the same unique household-person identifiers creating respondent-record observations. All CCHS respondents were retained. If a respondent had no hospitalizations, then hospitalization variable values were set to missing.

#### Study population

According to information provided at time of interview, the following exclusion criteria were applied to the cohort of CCHS respondents who agreed to share and link their survey data to health administrative data in order of decreasing frequency of respondents removed at each step: 1) Age < 18 years or age > 74 years; 2) Quebec residence; 3) Missing household income quintile; 4) Missing material deprivation quintile; and 5) Pregnant status.

The study population was limited to adults between the ages of 18-74 years of age for two reasons. First, the lower age limit was applied to facilitate interpretation of SES in adulthood which differs from SES in youth who are generally under parental or legal guardian care, have not completed their education, and are not yet employed in career occupations. Second, the upper age limit was consistent with the Canadian Institute of Health Information definition of ACSC hospitalizations and implies that hospitalizations for these conditions after the age of 74 years are either less avoidable or completely unavoidable given decreased health status of older individuals [2]. Quebec residents were excluded as their survey data could not be linked to hospital separation records. Respondents with missing primary exposure information were excluded as the effect of SES on risk of ACSC hospitalization could not be studied in these individuals. To mitigate the number of respondents excluded due to missing income information, data files with imputed household income information provided by Statistics Canada for survey cycles 2005-2010 were used. Missing material deprivation information was either due to an inability to match postal code information to a DA identifier or deprivation data was missing for a given DA in CanMarg. Pregnant women were excluded as some baseline covariates used in this study may have been misclassified due to their pregnancy status (e.g. temporarily quitting smoking or consuming alcohol). Lastly, respondents with recording errors (e.g. in-hospital death date prior to CCHS interview date) were also excluded. 

### Variable definitions

#### Outcome variable

The primary outcome variable was risk of index hospitalization with a primary diagnosis of one of the following chronic ACSCs: Angina (without select cardiac interventions), asthma, CHF (without select cardiac interventions), COPD, diabetes and select diabetic complications, epilepsy, and hypertension (without select cardiac interventions), where the respondent was between 18-74 years of age at time of admission, admitted to an acute care institution, and alive at discharge. International Classification of Diseases (ICD) -9, ICD-9-CM and ICD-10 diagnostic codes and intervention codes were consistent with the CIHI definition of ACS conditions. Outcome information was determined from the DAD.

#### Exposure variables

The primary exposure variable includes both joint individual-level national household income quintile and DA-level material deprivation quintile. Specifically, national household income quintiles were derived from household income information provided by each respondent in the CCHS. National material deprivation quintiles were derived from SES information at the DA level. National household income quintile and material deprivation quintile were ascertained from the CCHS and CanMarg, respectively. Income quintiles 1-2 were classified as “low income” and quintiles 3-5 as “high income”. Material deprivation quintiles 1-3 were classified as “low deprivation” and quintiles 4-5 as “high deprivation”. Based on this classification scheme, four categorical levels were created: Low income-low deprivation, low income-high deprivation, high income-high deprivation, and high income-low deprivation.

#### Covariates

Covariates were determined from the CCHS and included demographic variables (age, sex, self-identified ethnicity, rurality), socioeconomic variables (highest level of education in a respondent’s household, marital status, immigrant status), behavioural variables (type of smoker, alcohol consumption in past year, body mass index (BMI), and level of physical activity), and survey cycle. All variables were modeled as categorical variables except age which was modelled as a restrictive cubic spline with five knots at the 5^th^, 27.5^th^, 50^th^, 72.5^th^, and 95^th^ percentiles. BMI values were corrected for potential misclassification of height and weight information [26]. Missing household education information was imputed using individual education information if known.

### Analysis

Descriptive statistics (proportions, means) were generated and stratified by type of prospective hospitalization. Three mutually exclusive categories of type of prospective hospitalization were created: avoidable, unavoidable, and none. First, respondents with an avoidable hospitalization were categorized as avoidable. Of the remaining respondents, respondents who experienced a hospitalization for a non-ACSC condition or were hospitalized after age 74 years for any condition were categorized as unavoidable. Respondents who were never hospitalized (i.e. no DAD records) or were only hospitalized before their interview were categorized as none.

Relative risk was estimated by constructing modified Poisson regression models with robust error variance using a binary count variable (1 = Experienced at least one prospective avoidable hospitalization (i.e. avoidable respondents); 0 = Did not experience an avoidable hospitalization (i.e. unavoidable and none respondents) and logged observation time as the offset variable for respondents with complete information [27]. Observation time was calculated as the time since interview to the first of the following events: 1) Index ACSC hospitalization; 2) In-hospital death; 3) 75th birthdate; 4) End of study (March 31, 2013). Models were sequentially adjusted following a conceptual model that moves from demographic to socioeconomic to behavioural factors. We present these sequentially adjusted models as follows: 1) Age, sex, and survey cycle (Model 1); 2) Addition of demographic variables self-identified ethnicity (white/non-white) and rurality (urban/rural) (Model 2); 3) Addition of socioeconomic variables marital status (single/married or common-law/separated or divorced/widowed), immigrant status (domestic/immigrant), and highest level of household education (less than secondary/secondary completed/some post-secondary/post-secondary completed) (Model 3); and, 4) Addition of behavioural variables type of smoker (daily/occasional/former daily/former occasional/never), alcohol consumption in the past year (regular/occasional/never), BMI (obese (≥ 30 kg/m2)/overweight (25 – 29.9 kg/m2)/normal (18.5 – 24.9 kg/m2)/underweight (< 18.5 kg/m2)), and physical activity (active/moderate/inactive). A pooled (scaled) weight variable was constructed by dividing the share-link weight associated with each respondent by the number of pooled CCHS cycles [28]. A normalized weight variable was then generated by dividing the pooled weight variable by the study cohort pooled weight mean. Models were weighted using the normalized weight variable.

We conducted four sensitivity analyses to test the robustness of the findings. First, to reduce the potential for reverse causation, a one-year washout period was used whereby any ACSC hospitalizations during the first 365 days after the interview date were not counted as events. Second, we analyzed a sub-cohort of respondents that excluded those with a prospective unavoidable hospitalization such that an outcome value of zero represented no prospective hospitalizations to test the impact of our choice of classification. Third, classification of quintile 3 as high income and low deprivation in the joint exposure variable was evaluated by re-classifying quintile 3 as low income and high deprivation to test the impact of our categorization. Finally, use of robust error variance to construct 95% confidence intervals and a normalized model weight variable that do not account for the CCHS complex survey design was assessed by constructing logistic models with 95% confidence intervals calculated using balanced repeated replication and scaled bootstrap weight variables and a pooled model weight variable.

All data was accessed and analyzed in a Statistics Canada Research Data Centre (RDC) at the University of Toronto and University of Guelph following submission and approval of a project proposal by Statistics Canada. RDCs facilitate access to Statistics Canada microdata in secure computing environments at participating Canadian universities. Additional information on RDCs and the application process can be found at https://www.statcan.gc.ca/eng/microdata/data-centres. All analyses were conducted using SAS Version 9.4.

## Results

### Cohort creation

**Figure 1: Study flow diagram:**
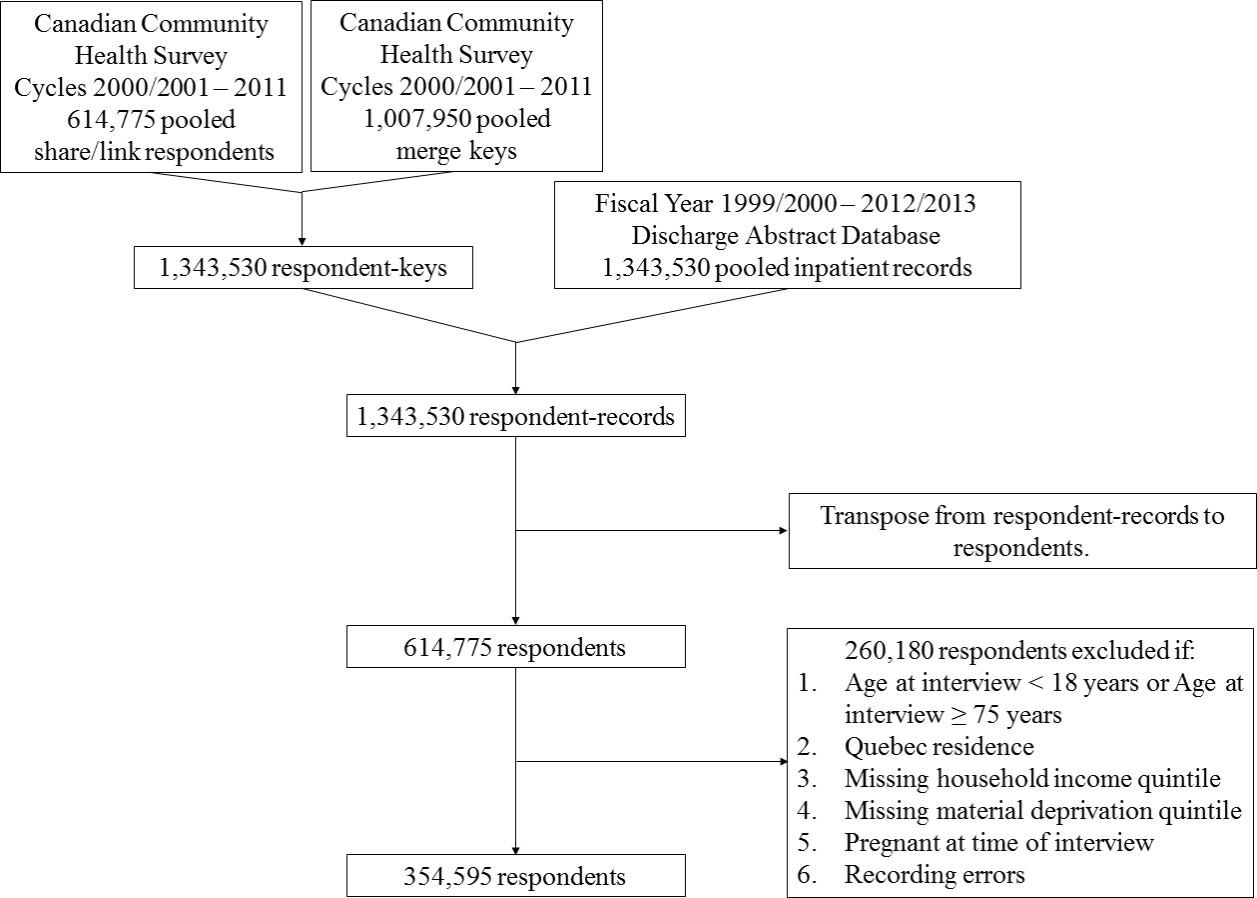


After applying exclusion criteria to the initial cohort of 614,775 pooled CCHS respondents, there were 354,595 respondents in the study cohort (Figure 1). There were no duplicate respondents.

### Descriptive statistics

Of these respondents, 7,430 respondents experienced at least one avoidable hospitalization. These respondents tended to have lower individual-level household income and live in more materially deprived neighbourhoods compared to respondents who experienced at least one unavoidable hospitalization (n=123,640) or no hospitalizations during follow-up time (n=223,525) (Table 1).

**Table 1: Socioeconomic exposures of pooled CCHS respondents (cycles 2000/2001 - 2011) with complete exposure information stratified by type of prospective hospitalization (n = 354,595).** 

	Avoidable hospitalization	Unavoidable hospitalization	No hospitalization;
	(n = 7,430)	(n = 123,640)	(n = 223,525)
Characteristic	Percentage	Percentage	Percentage
Individual-level national household income quintile			
Q1 (Lowest income)	29.0	18.1	16.7
Q2	25.0	18.8	17.7
Q3	16.0	19.7	20.2
Q4	15.5	20.5	21.3
Q5 (Highest income)	15.0	22.8	24.0
DA-level national material deprivation quintile			
Q5 (Most deprived)	24.0	17.6	13.7
Q4	20.5	18.4	17.4
Q3	21.0	20.2	19.8
Q2	19.5	21.5	22.5
Q1 (Least deprived)	14.5	22.3	26.6
Joint income-deprivation			
Low income (Q1-Q2) - High deprivation (Q4-Q5)	28.0	17.9	15.0
Low income (Q1-Q2) - Low deprivation (Q1-Q3)	26.0	19.0	19.4
High income (Q3-Q5) - High deprivation (Q4-Q5)	16.5	18.0	16.1
High income (Q3-Q5) - Low deprivation (Q1-Q3)	29.5	45.0	49.5

### Main results

Considering individual-level household income quintile and neighbourhood-level material deprivation quintile as separate exposures, in age-, sex-, and cycle-adjusted models, risk of avoidable hospitalization increased in a graded manner across both income quintiles and deprivation quintiles (Model 1) (Table 2). Adjustment for demographic variables slightly increased income effect sizes but had no effect on deprivation effect sizes (Model 2). Additional adjustment for other socioeconomic variables attenuated effect sizes, particularly for income quintiles 1 and 2 and deprivation quintiles 3-5 (Model 3). Here, individuals in the lowest income quintile and those living in the most deprived neighbourhoods were more than twice as likely to experience an avoidable hospitalization relative to those in the highest income quintile and living in the least deprived neighbourhoods, respectively. Final adjustment for behavioural variables further attenuated the effects of household income and material deprivation on risk of avoidable hospitalization (Income: RR 1.82 (1.56-2.13) Deprivation: RR 1.67 (1.44-1.95)) (Model 4). When both individual-level household income quintile and neighbourhood-level material deprivation quintile were entered in the model together, a similar pattern was observed with more attenuated effect sizes relative to the single exposure models.

**Table 2: Multivariable Sequentially Adjusted Modified Poisson Regression Models with Robust Error Variance for Individual-Level National Household Income Quintile, DA-Level National Material Deprivation Quintile, and Index Prospective ACSC Hospitalization for Pooled CCHS Respondents (Cycles 2000/2001-2011) (N= 335,845).** ^a^Adjusted for age, sex, and survey cycle ^b^Adjusted for age, sex, self-identified ethnicity, rurality, and survey cycle ^c^Adjusted for age, sex, self-identified ethnicity, rurality, marital status, immigrant status, highest level of household education, and survey cycle ^d^Adjusted for age, sex, self-identified ethnicity, rurality, marital status, immigrant status, highest level of household education, smoking, alcohol consumption, body mass index, physical activity, and survey cycle

Exposure: Individual-level national household income quintile				

	MODEL 1a	MODEL 2b	MODEL 3c	MODEL 4d
	RR (95% CI)	RR (95% CI)	RR (95% CI)	RR (95% CI)
Q1 (Lowest income)	2.76	3.1	2.66	1.82
	(2.41 - 3.17)	(2.69 - 3.58)	(2.29 - 3.09)	(1.56 - 2.13)
Q2	2.1	2.24	2.09	1.62
	(1.80 - 2.44)	(1.93 - 2.60)	(1.78 - 2.44)	(1.38 - 1.90)
Q3	1.28	1.34	1.28	1.08
	(1.11 - 1.47)	(1.17 - 1.55)	(1.11 - 1.48)	(0.93 - 1.25)
Q4	1.23	1.26	1.24	1.13
	(1.06 - 1.44)	(1.08 - 1.47)	(1.07 - 1.45)	(0.97 - 1.33)
Q5 (Highest income)	1	1	1	1

Exposure: DA-level national material deprivation quintile				

Q5 (Most deprived)	2.74	2.74	2.27	1.67
	(2.36 - 3.17)	(2.37 - 3.18)	(1.95 - 2.63)	(1.44 - 1.95)
Q4	1.96	1.97	1.74	1.43
	(1.69 - 2.27)	(1.70 - 2.28)	(1.50 - 2.02)	(1.22 - 1.66)
Q3	1.73	1.72	1.6	1.37
	(1.47 - 2.03)	(1.46 - 2.02)	(1.35 - 1.88)	(1.16 - 1.62)
Q2	1.47	1.45	1.4	1.28
	(1.25 - 1.73)	(1.23 - 1.70)	(1.19 - 1.65)	(1.08 - 1.51)
Q1 (Least deprived)	1	1	1	1

Exposures: National household income quintile and material deprivation quintile				

National household income quintile				
Q1 (Lowest income)	2.28	2.57	2.33	1.69
	(1.98 - 2.63)	(2.22 - 2.97)	(2.00 - 2.71)	(1.44 - 1.98)
Q2	1.82	1.95	1.89	1.53
	(1.56 - 2.13)	(1.68 - 2.27)	(1.61 - 2.21)	(1.30 - 1.79)
Q3	1.17	1.23	1.2	1.04
	(1.01 - 1.34)	(1.07 - 1.41)	(1.04 - 1.38)	(0.90 - 1.20)
Q4	1.17	1.2	1.19	1.1
	(1.00 - 1.36)	(1.03 - 1.39)	(1.02 - 1.39)	(0.94 - 1.29)
Q5 (Highest income)	1	1	1	1
National material deprivation quintile				
Q5 (Most deprived)	2.15	2.09	1.88	1.5
	(1.84 - 2.50)	(1.80 - 2.44)	(1.61 - 2.19)	(1.28 - 1.76)
Q4	1.67	1.66	1.55	1.34
	(1.44 - 1.94)	(1.43 - 1.93)	(1.34 - 1.80)	(1.14 - 1.56)
Q3	1.55	1.52	1.47	1.31
	(1.32 - 1.82)	(1.30 - 1.79)	(1.25 - 1.73)	(1.11 - 1.55)
Q2	1.39	1.36	1.34	1.25
	(1.18 - 1.63)	(1.15 - 1.59)	(1.14 - 1.58)	(1.06 - 1.47)
Q1 (Least deprived)	1	1	1	1

When individual- and neighbourhood-level SES variables were considered jointly, low-income individuals in both low and high material deprivation neighbourhoods had more than double the risk of an avoidable hospitalization after adjusting for demographic and socioeconomic variables, with individuals living in high material deprivation neighbourhoods at greatest risk (Models 1-3) (Table 3). This effect was attenuated after adjusting for behavioural variables (Model 4). For low-income individuals, living in more deprived neighbourhoods increased risk of avoidable hospitalization, although the difference was less pronounced after full adjustment. For high-income individuals, living in more deprived neighbourhoods similarly increased the risk of avoidable hospitalization, even after full adjustment (RR 1.34 (1.19 - 1.52)).

**Table 3: Multivariable Sequentially Adjusted Modified Poisson Regression Models with Robust Error Variance for Joint Individual-Level National Household Income Quintile and DA-Level National Material Deprivation Quintile Exposure and Index Prospective ACSC Hospitalization for Pooled CCHS Respondents (Cycles 2000/2001-2011) (N =335,845):** ^a^Adjusted for age, sex, and survey cycle ^b^Adjusted for age, sex, self-identified ethnicity, rurality, and survey cycle ^c^Adjusted for age, sex, self-identified ethnicity, rurality, marital status, immigrant status, highest level of household education, and survey cycle ^d^Adjusted for age, sex, self-identified ethnicity, rurality, marital status, immigrant status, highest level of household education, smoking, alcohol consumption, body mass index, physical activity, and survey cycle

	MODEL 1a	MODEL 2b	MODEL 3c	MODEL 4d
	RR (95% CI)	RR (95% CI)	RR (95% CI)	RR (95% CI)
Joint income-deprivation				

Low income (Q1-Q2) - High deprivation (Q4-Q5)	2.81	2.99	2.54	1.83
	(2.54 - 3.12)	(2.70 - 3.32)	(2.28 - 2.84)	(1.63 - 2.05)
Low income (Q1-Q2) - Low deprivation (Q1-Q3)	2.1	2.2	2.08	1.71
	(1.84 - 2.40)	(1.94 - 2.51)	(1.82 - 2.39)	(1.49 - 1.96)
High income (Q3-Q5) - High deprivation (Q4-Q5)	1.66	1.64	1.55	1.34
	(1.48 - 1.87)	(1.45 - 1.84)	(1.37 - 1.75)	(1.19 - 1.52)
High income (Q3-Q5) - Low deprivation (Q1-Q3)	1	1	1	1

Likewise, for individuals living in low deprivation neighbourhoods, decreased household income doubled the risk of avoidable hospitalization after adjusting for demographic and socioeconomic variables with some attenuation of effect after adjusting for behavioural variables. Similarly, for those living in high deprivation neighbourhoods, decreased income also increased risk of hospitalization after full adjustment, although this effect was less pronounced.

### Sensitivity analyses

Overall, our analysis was robust to a series of planned sensitivity analyses. In our analysis with the one-year washout period, we found the results did not change and thus less likely affected by reverse causation. (Supplementary Appendix 1). We also found findings were robust to the way the outcome variable was operationalized. Specifically, excluding individuals with an unavoidable hospitalization did not affect overall patterns, however as reasonably expected, effect sizes did slightly increase relative to the original joint exposure model (Supplementary Appendix 2). When looking at an alternative way to collapse the third income quintile, we found that re-categorizing quintile 3 as low income and high deprivation similarly did not affect overall patterns but did attenuate effect sizes (Supplementary Appendix 3). Lastly, accounting for the complex survey design using robust error variation did not appreciably alter 95% confidence interval widths relative to robust variance estimation in the single material deprivation, single income and material deprivation, and joint exposure models (Supplementary Appendix 4).

## Discussion

This is the first national-level study of both individual- and neighbourhood-level SES on risk of avoidable hospitalizations in Canada using linked health survey and administrative data and a population data science approach [29]. We examine this association in various ways including controlling for potential individual-level confounders and accounting for time-at-risk in our analysis. The results demonstrate that individual and neighbourhood SES separately and jointly increase risk of avoidable hospitalization. Low income individuals living in high deprivation neighbourhoods were at greatest risk of hospitalization. However, both high income individuals in high deprivation neighbourhoods and low income individuals in low deprivation neighbourhoods were also at increased risk suggesting that both individual and neighbourhood SES contribute to risk, even after controlling for potential individual-level confounders.

Prior studies have demonstrated that individual and neighbourhood SES separately increase risk of avoidable hospitalizations for aggregate and disease-specific conditions, [9, 13-18, 30-32] while others found non-significant attenuated effects after full adjustment of their models [33-35]. Differential selection and control of confounding variables, heterogeneity in exposure and outcome definitions, and use of various modelling approaches may all contribute to variation in findings from prior studies. However, most studies confirm that low SES is associated with a higher risk of avoidable hospitalizations in different geographic locations and patient populations.

There are a number of potential mechanisms by which lower SES increases risk of avoidable hospitalizations at both the health system and individual levels, despite universal health care in Canada [36]. At the health system level, individuals of lower SES may experience disparities in primary care that hinder ongoing management of chronic conditions and increase risk of hospitalization [37]. Previous work suggests that lower SES individuals are able to access health care; however, also may be less likely to receive appropriate care needed to manage their conditions [37]. In addition, individuals with lower SES may have greater access to acute care services versus primary care [38]. Health literacy may also be playing a role [39]. For example, health literacy mediated the association between education and hypertension knowledge and control [40], glycemic control [41], and self-rated health [42]. The increased burden of health behaviours, such as smoking, may also be contributing [43-45]. For example, health behaviours mediated part of the relationship between SES and type 2 diabetes incidence among an Australian adult cohort [46]. Specific pathways from SES to risk of avoidable hospitalizations has not been well studied and represents an important area of research needed to design effective policy and interventions.

This study has a number of strengths that overcomes some prior limitations in previous studies. First, we used household income for each CCHS respondent, expressed as national household income quintiles as well as neighbourhood material deprivation quintiles according to each respondent’s postal code. We have shown that area-based and individual-level income measures do not agree well in this study population, and both measures should be considered to provide more socioeconomic information relative to each measure alone [47]. The use of individual-level income information overcomes potential ecological bias that can occur when using neighbourhood-level measures to make individual-level inferences. The additional use of neighbourhood-level material deprivation allowed for estimation of both separate and joint effects. Joint SES effects on avoidable hospitalizations are not well understood with this study contributing important results to address this knowledge gap. Second, sequentially adjusted modified Poisson regression models were used and reported allowing for more accurate and transparent estimation of effects, accounting for individual time at risk. Sensitivity analyses of various modelling decisions made in this study confirmed that the overall pattern, magnitude, and significance of results were robust. Third, this study used a large, national cohort of individuals with rich demographic, socioeconomic, and health behavioural information allowing for adjustment of important confounders when estimating the impact of SES on risk of avoidable hospitalizations.

This study also had several limitations that are important to acknowledge. The study population was limited to CCHS respondents who agreed to share and link their data (>80% of CCHS respondents) and had complete household income and material deprivation information. Although there are minor differences between the full CCHS cohort and those who agreed to share and link their data, share/link survey weights developed by Statistics Canada were used in this study to account for these differences [48]. Available imputed household income from Statistics Canada was used for cycles 2005-2010 to reduce the number of individuals with missing income information. Linkage of material deprivation information was limited to available 2001 and 2006 CanMarg datasets. These datasets are not complete and do have missing information for certain DAs. For respondents from later CCHS cycles, it is possible that 2006 neighbourhood deprivation did not accurately represent their baseline neighbourhood SES status if neighbourhood SES changed over time. Use of broader deprivation categories should minimize potential misclassification bias as larger changes in neighbourhood SES would likely be needed to change quintiles or change between low and high deprivation groups. Lastly, estimation of time at risk was incomplete as we were unable to censor individuals who died outside of hospital, overestimating their time at risk. Given that this study focused on the adult population, aged 18-74 years, with a relatively low mortality rate (e.g. compared to elderly populations), this is unlikely to have a large effect.

These study results are comparable to other studies of general adult populations with access to a health system similar to the Canadian health system, which is a universal health care system, and that use similar income and deprivation exposures. Caution should be used when extending these results to studies employing different definitions (e.g. avoidable hospitalizations for acute, chronic, and vaccine-preventable conditions). This study did not report results by condition as the objective was not to estimate condition-specific effects given the small number of events within disease categories. The composite measure is also more consistent with the use of avoidable hospitalization as a health system indicator in Canada [2].

## Conclusion

In conclusion, this study demonstrates the joint effect of individual income and neighbourhood deprivation on risk of avoidable hospitalizations using a large, linked database of health survey and administrative information. Both household income and neighbourhood deprivation contribute to rates of unnecessary hospitalization. Future work could examine other dimensions of SES to generate a more nuanced understanding of the role of SES in determining risk of avoidable hospitalization. Both individual- and neighbourhood-level SES should be considered when designing programs and policies to reduce avoidable hospitalizations in Canada.

## Ethics statement

This study was approved by the University of Toronto Research Ethics Board (Protocol 37499).

## Supplementary Appendices

Supplementary Appendix 1 - Multivariable Sequentially Adjusted Modified Poisson Regression Models with Robust Error Variance for Individual-Level National Household Income Quintile, DA-Level National Material Deprivation Quintile, and Joint Income-Deprivation and Index Prospective ACSC Hospitalization Excluding Hospitalizations Occurring within the First Year from Time of Interview for Pooled CCHS Respondents (Cycles 2000/2001-2011) (N = 335,845)

Supplementary Appendix 2 - Multivariable Sequentially Adjusted Modified Poisson Regression Models with Robust Error Variance for Joint Individual-Level National Household Income Quintile and DA-Level National Material Deprivation Quintile Exposure and Index Prospective ACSC Hospitalization Comparing Respondents with an Avoidable Hospitalization to Respondents with No Prospective Hospitalization for Pooled CCHS Respondents (Cycles 2000/2001-2011) (N = 218,795)

Supplementary Appendix 3 - Multivariable Sequentially Adjusted Modified Poisson Regression Models with Robust Error Variance for Joint Individual-Level National Household Income Quintile and DA-Level National Material Deprivation Quintile Exposure Categorizing Quintile 3 as Low Income and High Deprivation and Index Prospective ACSC Hospitalization for Pooled CCHS Respondents (Cycles 2000/2001 - 2011) (N = 335,845)

Supplementary Appendix 4 - Multivariable Sequentially Adjusted Logistic Regression Models with Bootstrap Variance for Individual-Level National Household Income Quintile, DA-Level National Material Deprivation Quintile, and Joint Income-Deprivation and Index Prospective ACSC Hospitalization Excluding Hospitalizations Occurring within the First Year from Time of Interview for Pooled CCHS Respondents (Cycles 2000/2001-2011) (N = 335,845)

**Supplementary Appendix 1. Multivariable Sequentially Adjusted Modified Poisson Regression Models with Robust Error Variance for Individual-Level National Household Income Quintile, DA-Level National Material Deprivation Quintile, and Joint Income-Deprivation and Index Prospective ACSC Hospitalization Excluding Hospitalizations Occurring within the First Year from Time of Interview for Pooled CCHS Respondents (Cycles 2000/2001-2011) (N = 335,845):** ^a^Adjusted for age, sex, and survey cycle ^b^Adjusted for age, sex, self-identified ethnicity, rurality, and survey cycle ^c^Adjusted for age, sex, self-identified ethnicity, rurality, marital status, immigrant status, highest level of household education, and survey cycle ^d^Adjusted for age, sex, self-identified ethnicity, rurality, marital status, immigrant status, highest level of household education, smoking, alcohol consumption, body mass index, physical activity, and survey cycle

Exposure: Individual-level national household income quintile				

	MODEL 1a	MODEL 2b	MODEL 3c	MODEL 4d
	RR (95% CI)	RR (95% CI)	RR (95% CI)	RR (95% CI)
Q1 (Lowest income)	2.71	3.01	2.53	1.74
	(2.33 - 3.14)	(2.59 - 3.51)	(2.15 - 2.97)	(1.47 - 2.06)
Q2	2.11	2.24	2.07	1.6
	(1.78 - 2.50)	(1.90 - 2.64)	(1.74 - 2.46)	(1.35 - 1.91)
Q3	1.28	1.34	1.28	1.09
	(1.10 - 1.49)	(1.15 - 1.57)	(1.09 - 1.49)	(0.93 - 1.28)
Q4	1.23	1.26	1.24	1.13
	(1.04 - 1.45)	(1.07 - 1.49)	(1.05 - 1.47)	(0.95 - 1.34)
Q5 (Highest income)	1	1	1	1

Exposure: DA-level national material deprivation quintile				

Q5 (Most deprived)	2.61	2.62	2.15	1.58
	(2.22 - 3.07)	(2.23 - 3.08)	(1.82 - 2.53)	(1.34 - 1.87)
Q4	1.84	1.85	1.63	1.33
	(1.57 - 2.17)	(1.58 - 2.18)	(1.39 - 1.92)	(1.12 - 1.57)
Q3	1.61	1.61	1.49	1.29
	(1.35 - 1.93)	(1.34 - 1.92)	(1.24 - 1.78)	(1.07 - 1.55)
Q2	1.41	1.38	1.34	1.21
	(1.18 - 1.68)	(1.15 - 1.65)	(1.12 - 1.60)	(1.01 - 1.45)
Q1 (Least deprived)	1	1	1	1

Exposures: National household income quintile and material deprivation quintile				

National household income quintile				
Q1 (Lowest income)	2.26	2.51	2.23	1.62
	(1.94 - 2.63)	(2.15 - 2.94)	(1.89 - 2.63)	(1.37 - 1.93)
Q2	1.85	1.97	1.88	1.52
	(1.56 - 2.20)	(1.67 - 2.33)	(1.59 - 2.24)	(1.28 - 1.81)
Q3	1.18	1.23	1.2	1.05
	(1.01 - 1.37)	(1.06 - 1.44)	(1.03 - 1.40)	(0.90 - 1.23)
Q4	1.17	1.2	1.2	1.1
	(0.99 - 1.38)	(1.02 - 1.42)	(1.01 - 1.41)	(0.93 - 1.31)
Q5 (Highest income)	1	1	1	1
National material deprivation quintile				
Q5 (Most deprived)	2.05	2.01	1.8	1.43
	(1.74 - 2.42)	(1.70 - 2.38)	(1.52 - 2.12)	(1.20 - 1.70)
Q4	1.58	1.57	1.46	1.25
	(1.34 - 1.86)	(1.33 - 1.85)	(1.24 - 1.72)	(1.05 - 1.48)
Q3	1.45	1.43	1.37	1.24
	(1.21 - 1.73)	(1.19 - 1.70)	(1.14 - 1.64)	(1.03 - 1.49)
Q2	1.33	1.29	1.28	1.18
	(1.11 - 1.59)	(1.08 - 1.55)	(1.07 - 1.53)	(0.98 - 1.41)
Q1 (Least deprived)	1	1	1	1

Exposure: Joint income-deprivation

Low income (Q1-Q2) - High deprivation (Q4-Q5)	2.76	2.93	2.45	1.75
	(2.47 - 3.09)	(2.62 - 3.28)	(2.18 - 2.76)	(1.55 - 1.98)
Low income (Q1-Q2) - Low deprivation (Q1-Q3)	2.08	2.17	2.02	1.67
	(1.79 - 2.41)	(1.88 - 2.50)	(1.74 - 2.35)	(1.43 - 1.95)
High income (Q3-Q5) - High deprivation (Q4-Q5)	1.63	1.61	1.52	1.33
	(1.43 - 1.86)	(1.41 - 1.84)	(1.33 - 1.73)	(1.16 - 1.52)
High income (Q3-Q5) - Low deprivation (Q1-Q3)	1	1	1	1

**Supplementary Appendix 2. Multivariable Sequentially Adjusted Modified Poisson Regression Models with Robust Error Variance for Joint Individual-Level National Household Income Quintile and DA-Level National Material Deprivation Quintile Exposure and Index Prospective ACSC Hospitalization Comparing Respondents with an Avoidable Hospitalization to Respondents with No Prospective Hospitalization for Pooled CCHS Respondents (Cycles 2000/2001-2011) (N = 218,795):** ^a^Adjusted for age, sex, and survey cycle ^b^Adjusted for age, sex, self-identified ethnicity, rurality, and survey cycle ^c^Adjusted for age, sex, self-identified ethnicity, rurality, marital status, immigrant status, highest level of household education, and survey cycle ^d^Adjusted for age, sex, self-identified ethnicity, rurality, marital status, immigrant status, highest level of household education, smoking, alcohol consumption, body mass index, physical activity, and survey cycle

	MODEL 1a	MODEL 2b	MODEL 3c	MODEL 4d
	RR (95% CI)	RR (95% CI)	RR (95% CI)	RR (95% CI)
Joint income-deprivation				

Low income (Q1-Q2) - High deprivation (Q4-Q5)	2.97	3.27	2.81	1.99
	(2.68 - 3.30)	(2.94 - 3.62)	(2.52 - 3.14)	(1.78 - 2.24)
Low income (Q1-Q2) - Low deprivation (Q1-Q3)	2.11	2.27	2.16	1.76
	(1.85 - 2.41)	(2.00 - 2.59)	(1.88 - 2.48)	(1.53 - 2.02)
High income (Q3-Q5) - High deprivation (Q4-Q5)	1.81	1.78	1.68	1.45
	(1.60 - 2.04)	(1.58 - 2.01)	(1.49 - 1.90)	(1.28 - 1.64)
High income (Q3-Q5) - Low deprivation (Q1-Q3)	1	1	1	1

**Supplementary Appendix 3. Multivariable Sequentially Adjusted Modified Poisson Regression Models with Robust Error Variance for Joint Individual-Level National Household Income Quintile and DA-Level National Material Deprivation Quintile Exposure Categorizing Quintile 3 as Low Income and High Deprivation and Index Prospective ACSC Hospitalization for Pooled CCHS Respondents (Cycles 2000/2001 - 2011) (N = 335,845):** ^a^Adjusted for age, sex, and survey cycle ^b^Adjusted for age, sex, self-identified ethnicity, rurality, and survey cycle ^c^Adjusted for age, sex, self-identified ethnicity, rurality, marital status, immigrant status, highest level of household education, and survey cycle ^d^Adjusted for age, sex, self-identified ethnicity, rurality, marital status, immigrant status, highest level of household education, smoking, alcohol consumption, body mass index, physical activity, and survey cycle

	MODEL 1a	MODEL 2b	MODEL 3c	MODEL 4d
	RR (95% CI)	RR (95% CI)	RR (95% CI)	RR (95% CI)
Joint income-deprivation				

Low income (Q1-Q2) - High deprivation (Q4-Q5)	2.51	2.62	2.24	1.62
	(2.19 - 2.87)	(2.30 - 3.00)	(1.94 - 2.58)	(1.40 - 1.88)
Low income (Q1-Q2) - Low deprivation (Q1-Q3)	1.57	1.62	1.53	1.28
	(1.33 - 1.84)	(1.38 - 1.90)	(1.30 - 1.80)	(1.08 - 1.52)
High income (Q3-Q5) - High deprivation (Q4-Q5)	1.47	1.45	1.38	1.22
	(1.26 - 1.72)	(1.24 - 1.69)	(1.18 - 1.61)	(1.04 - 1.43)
High income (Q3-Q5) - Low deprivation (Q1-Q3)	1	1	1	1

**Supplementary Appendix 4. Multivariable Sequentially Adjusted Logistic Regression Models with Bootstrap Variance for Individual-Level National Household Income Quintile, DA-Level National Material Deprivation Quintile, and Joint Income-Deprivation and Index Prospective ACSC Hospitalization Excluding Hospitalizations Occurring within the First Year from Time of Interview for Pooled CCHS Respondents (Cycles 2000/2001-2011) (N = 335,845):** ^a^Adjusted for age, sex, and survey cycle ^b^Adjusted for age, sex, self-identified ethnicity, rurality, and survey cycle ^c^Adjusted for age, sex, self-identified ethnicity, rurality, marital status, immigrant status, highest level of household education, and survey cycle ^d^Adjusted for age, sex, self-identified ethnicity, rurality, marital status, immigrant status, highest level of household education, smoking, alcohol consumption, body mass index, physical activity, and survey cycle

Exposure: Individual-level national household income quintile				

	MODEL 1a	MODEL 2b	MODEL 3c	MODEL 4d
	RR (95% CI)	RR (95% CI)	RR (95% CI)	RR (95% CI)
Q1 (Lowest income)	2.83	3.19	2.74	1.87
	(2.45 - 3.27)	(2.75 - 3.71)	(2.34 - 3.22)	(1.58 - 2.21)
Q2	2.16	2.31	2.16	1.67
	(1.84 - 2.53)	(1.98 - 2.70)	(1.84 - 2.54)	(1.42 - 1.97)
Q3	1.3	1.37	1.31	1.1
	(1.12 - 1.52)	(1.18 - 1.60)	(1.12 - 1.53)	(0.94 - 1.30)
Q4	1.24	1.28	1.26	1.15
	(1.06 - 1.46)	(1.09 - 1.50)	(1.07 - 1.48)	(0.97 - 1.35)
Q5 (Highest income)	1	1	1	1

Exposure: DA-level national material deprivation quintile				

Q5 (Most deprived)	2.76	2.77	2.29	1.68
	(2.40 - 3.18)	(2.40 - 3.19)	(1.99 - 2.65)	(1.45 - 1.95)
Q4	1.97	1.98	1.76	1.43
	(1.71 - 2.27)	(1.72 - 2.27)	(1.53 - 2.02)	(1.23 - 1.66)
Q3	1.74	1.73	1.61	1.38
	(1.49 - 2.03)	(1.49 - 2.02)	(1.38 - 1.89)	(1.17 - 1.63)
Q2	1.48	1.45	1.41	1.28
	(1.26 - 1.74)	(1.24 - 1.71)	(1.20 - 1.66)	(1.08 - 1.52)
Q1 (Least deprived)	1	1	1	1

Exposures: National household income quintile and material deprivation quintile				

National household income quintile				
Q1 (Lowest income)	2.34	2.65	2.4	1.73
	(2.03 - 2.71)	(2.28 - 3.08)	(2.05 - 2.81)	(1.47 - 2.05)
Q2	1.88	2.02	1.95	1.58
	(1.61 - 2.20)	(1.73 - 2.35)	(1.66 - 2.29)	(1.34 - 1.86)
Q3	1.19	1.25	1.22	1.06
	(1.02 - 1.39)	(1.07 - 1.46)	(1.04 - 1.43)	(0.90 - 1.24)
Q4	1.18	1.21	1.21	1.12
	(1.01 - 1.38)	(1.03 - 1.42)	(1.03 - 1.42)	(0.95 - 1.32)
Q5 (Highest income)	1	1	1	1
National material deprivation quintile				
Q5 (Most deprived)	2.16	2.1	1.89	1.5
	(1.87 - 2.49)	(1.82 - 2.42)	(1.64 - 2.18)	(1.30 - 1.75)
Q4	1.67	1.66	1.55	1.34
	(1.45 - 1.93)	(1.44 - 1.91)	(1.35 - 1.79)	(1.15 - 1.55)
Q3	1.56	1.53	1.48	1.32
	(1.34 - 1.81)	(1.32 - 1.78)	(1.27 - 1.73)	(1.12 - 1.55)
Q2	1.4	1.36	1.35	1.25
	(1.19 - 1.64)	(1.16 - 1.60)	(1.15 - 1.58)	(1.06 - 1.48)
Q1 (Least deprived)	1	1	1	1

Exposure: Joint income-deprivation

Low income (Q1-Q2) - High deprivation (Q4-Q5)	2.86	3.05	2.6	1.86
	(2.57 - 3.18)	(2.74 - 3.39)	(2.32 - 2.91)	(1.65 - 2.09)
Low income (Q1-Q2) - Low deprivation (Q1-Q3)	2.14	2.25	2.13	1.74
	(1.89 - 2.44)	(1.99 - 2.56)	(1.86 - 2.43)	(1.52 - 2.00)
High income (Q3-Q5) - High deprivation (Q4-Q5)	1.67	1.64	1.55	1.34
	(1.47 - 1.88)	(1.45 - 1.86)	(1.37 - 1.76)	(1.18 - 1.53)
High income (Q3-Q5) - Low deprivation (Q1-Q3)	1	1	1	1

## Abbreviations

ACSC	Ambulatory care sensitive condition
BMI	Body mass index
CanMarg	Canadian Marginalization Index
CCHS	Canadian Community Health Survey
CHF	Congestive heart failure
COPD	Chronic obstructive pulmonary disease
DA	Dissemination area
DAD	Discharge Abstract Database
FY	Fiscal year
ICD	International Classification of Diseases
PCCF+	Postal Code Conversion Files Plus
RR	Relative risk
SES	Socioeconomic status
